# Compressive Behavior of Pultruded GFRP Boxes with Concentric Openings Strengthened by Different Composite Wrappings

**DOI:** 10.3390/polym14194095

**Published:** 2022-09-29

**Authors:** Ceyhun Aksoylu, Yasin Onuralp Özkılıç, Emrah Madenci, Alexander Safonov

**Affiliations:** 1 Department of Civil Engineering, Konya Technical University, Konya 42130, Turkey; 2 Department of Civil Engineering, Necmettin Erbakan University, Konya 42140, Turkey; 3 Center for Materials Technologies, Skolkovo Institute of Science and Technology, 121205 Moscow, Russia

**Keywords:** hole, composite materials, EBR method, fiber-reinforced materials, compressive, opening, pultruded GFRP, FRP wrapping

## Abstract

Web openings often need to be created in structural elements for the passage of utility ducts and/or pipes. Such web openings reduce the cross-sectional area of the structural element in the affected region, leading to a decrease in its load-carrying capacity and stiffness. This paper experimentally studies the effect of web openings on the response of pultruded fiber-reinforced polymer (PFRP) composite profiles under compressive loads. A number of specimens have been processed to examine the behavior of PFRP profiles strengthened with one or more web openings. The effects of the size of the web opening and the FRP-strengthening scheme on the structural performance of PFRP profiles with FRP-strengthened web openings have been thoroughly analyzed and discussed. The decrease in load-carrying capacity of un-strengthened specimens varies between 7.9% and 66.4%, depending on the diameter of the web holes. It is observed that the diameter of the hole and the type of CFRP- or GFRP-strengthening method applied are very important parameters. All CFRP- and GFRP-strengthening alternatives were successful in the PFRP profiles, with diameter-to-width (D/W) ratios between 0.29 and 0.68. In addition, the load-carrying capacity after reinforcements made with CFRP and GFRP increased by 3.1–30.2% and 1.7–19.7%, respectively. Therefore, the pultruded profiles with openings are able to compensate for the reduction in load-carrying capacity due to holes, up to a D/W ratio of 0.32. The capacity significantly drops after a D/W ratio of 0.32. Moreover, the pultruded profile with CFRP wrapping is more likely to improve the load-carrying capacity compared to other wrappings. As a result, CFRP are recommended as preferred composite materials for strengthening alternatives.

## 1. Introduction

In the civil engineering sector, due to developing material technology in recent years, pultrusion fiber-reinforced polymer (PFRP) profiles have been used increasingly in buildings, bridges, and especially sea-water structures exposed to corrosion [1]. PFRP profiles are preferred due to advantages such as their light weight, high strength, improved durability, corrosion resistance, ease of transportation, assembly speed, and non-magnetic/non-conductive properties [2]. 

Pultrusion is carried out by pulling glass, carbon, or aramid fibers through a guide and by precisely distributing them according to the cross section of the profile [3,4]. In the pultrusion method, off-axis wound fibers replace continuous filament batts to be drawn together with axial fiber roving, allowing the laminate to achieve a higher fiber volume fraction with high-quality control and lower defective (resin-rich regions) content [5,6,7,8,9,10,11]. Polymers are usually filled by being blended with solid particles such as minerals or glass. These filler materials can add benefits to the materials in terms of improved processing, cost reduction, density control, improved optical and thermal properties, thermal expansion control, flame retardancy, and improvements in magnetic and electrical characteristics, in addition to promoted mechanical properties, such as fatigue resistance, wear resistance, and hardening [12,13,14,15,16]. Polymeric matrix composite materials are commonly reinforced with fibers: either continuous long fibers or chopped short fibers [17]. Current widely used FRPs include glass FRP (GFRP) and carbon FRP (CFRP). The low cost, tensile strength, and high deformability of GFRP and the high cost, excellent mechanical and fatigue-resistant properties of CFRP are the main differences between them. Under the coupling influence of the service environment, the improvement in mechanical properties and fatigue damage of FRP bars has become a major safety concern for structural applications [18]. For new applications, severe and complex service environments such as high temperature, hydraulic pressure, and cyclic load coupling can be a major challenge for FRPs [19,20,21]. Thanks to the hybrid effect, the advantages of the high mechanical and corrosion resistance of CFRP and the low price and high deformation of GFRP are used together [22]. Hybrid FRPs are expected to have excellent fatigue and corrosion resistance compared to single GFRPs [23].

**Figure 1:**
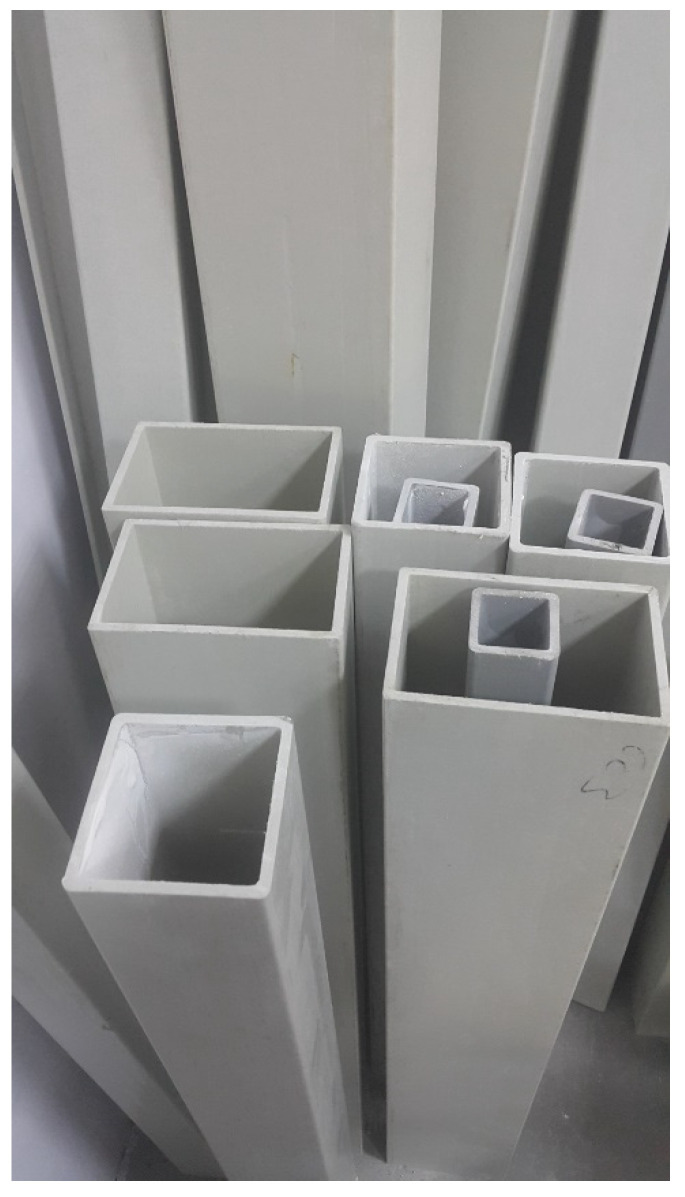
Pultruded GFRP profiles.

Currently, traditional materials such as concrete, steel, aluminum, and wood are rapidly losing their prevalence and are being replaced by highly efficient composite materials in many markets. PFRP beams also apply to the requirement to use web openings, often in beams made of conventional materials, to accommodate additional services when modifying and retrofitting existing buildings. Web openings often need to be created in PFRP beams and columns for the passage of utility ducts and/or pipes (Figure 1). Preformed rectangular or circular openings have already been made in the webs of PFRP beams for the passages of electrical, heating, and water supply systems, as well as utility ducts/pipes such as air conditioning vents, telephone wires, internet cables, and sewer pipes. Such web openings reduce the cross-sectional area of the beam in the affected area, leading to a reduction in its load-carrying capacity and stiffness. Therefore, a FRP reinforcement system is often required to be applied around the web opening to secure the weakened beam. Depending on their size and location, web openings can pose a major challenge to the strength and stiffness of the PFRP composite beam. Reinforcement around an opening is often required to maintain the required performance of the floor joist; traditionally, this is performed by applying additional steel plates bolted or welded in steel materials, while this is performed with FRP reinforcement in PFRP profiles. The opening size not only affects the load-carrying capacity of the beam but also has a significant impact on its failure mode [24]. When the opening size is small, the plane’s cross-sectional assumption still applies to the cross sections in the opening region [25]. In general, both the shear and the moment capacities of the perforated sections may be readily assessed. However, the moment capacities of the tee-sections above and below the web openings under local moments are relatively difficult to evaluate in the presence of co-existing axial and shear forces due to a global bending action. For beams with multiple web openings, buckling of web posts may be critical when the openings are closely spaced. Moreover, additional deflection due to the presence of web openings should also be considered [26].

Most existing studies on the behavior of reinforced concrete (RC) beams with a web opening have chosen to use FRP reinforcement (e.g., externally bonded FRP or near surface mounted FRP) for the strengthening of the opening region [27,28]. FRP has been found to be effective for strengthening RC members in structures because of its excellent mechanical properties [29,30,31,32,33,34,35,36,37,38]. A number of studies have been conducted to investigate the behavior of RC beams with an FRP-strengthened web opening. However, limited research is available on FRP-upgraded PFRP beams with web openings. Elsanadedy et al. [39] used carbon and glass FRPs in order to prevent a reduction in the stiffness and strength of the beams with rectangular web openings at the support region. Abdalla et al. [40] investigated the effect of the amount and configuration of the FRP in strengthening RC beams with web openings in the shear zone. Experimentally and numerically, the strengthening of the opening zones in RC beams using FRP rods was tested by Pimanmas [41]. A planned opening in the RC beam can be taken care of at the design stage by providing extra rebars around it. The effect of CFRP strengthening for the shear-deficient reinforced concrete beams with openings under a vertical load was examined by Aksoylu et al. [37].

FRP reinforcement schemes can be implemented in different ways. The most used FRP schemes in this literature can be listed as “vertical side bonded FRP sheets/plates or on the two sides of the opening [42,43,44,45]; vertically bonded FRP U-jackets or on the two sides of the opening [39,46]; vertically bonded FRP complete wraps or on the two sides of the opening [47]; diagonal side bonded FRP sheets/plates near the corners of the opening [48]; horizontally bonded FRP sheets/plates on the side surfaces or on the top and bottom surfaces of the beam [49]; diagonal near-surface mounted FRP bars at the opening corners”. Nie et al. [50,51] conducted a parametric study on the optimal design of the FRP reinforcement system for a typical beam attenuated by the creation of a mesh opening. Comparing the different FRP reinforcement schemes, it has been suggested that FRP cladding be used in practice, with FRP sheets glued horizontally on both the upper and lower beams, and on both sides of the opening and/or the side surfaces of the upper and lower beams. 

The aim of this research is to experimentally study the effect of large circular web openings on the behavior of un-strengthened and strengthened PFRP beams. Studied parameters include the size of the opening and the strengthening scheme. A total of 51 web-opening PFRP beams with reinforced FRP were prepared and tested under compressive loads. 

## 2. Experimental Program

In this study, the compressive behavior of the pultruded box with openings was investigated through experimental methods. Opening sizes and the types of strengthening were considered as primary variables, while the location of the openings and number of openings were selected as secondary variables. The size of the pultruded boxes was 75 × 75 × 6 mm, and the length of the boxes was 150 mm. Square pultruded GFRP box sections produced by Fiber Reinforced Composites Company, Manisa, Turkey, were used in this study. The tubes were produced using a pultrusion process with vinyl ester resin and E-glass fiber reinforcement. The mechanical properties of these pultruded GFRP profiles are given in Table 1. 

**Figure 2:**
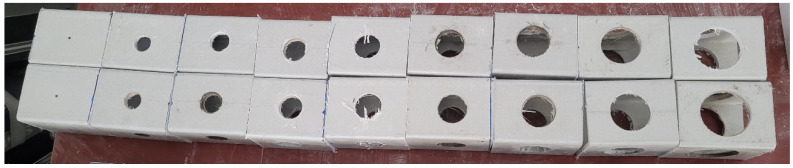
Specimens with different opening sizes.

Different hole sizes were opened in the test specimens. Hole sizes of 22 mm, 25 mm, 29 mm, 32 mm, 35 mm, 38 mm, 44 mm, and 51 mm were considered. The specimens without holes were also studied as a reference. The specimens with openings are shown in Figure 2 and were investigated. The specimens were strengthened with three different FRPs: single layer 800 gr/m^2^ 0° CFRP; 1200 gr/m^2^ 0° GFRP; and 300 g/m^2^ twill glass fiber fabric (GFRP) 0°/90°. For the FRP applications, F-1564 resin and F-3486-3487 hardener were used. Based on the manufacturer’s data, the resin and hardener mixing ratio was taken as 100/34 (by weight). The resin and hardener were carefully applied to achieve bonding between the pultruded box and FRP fabric. The material properties of FRP fabrics are given in Table 2. All specimens were fully wrapped.

**Figure 3:**
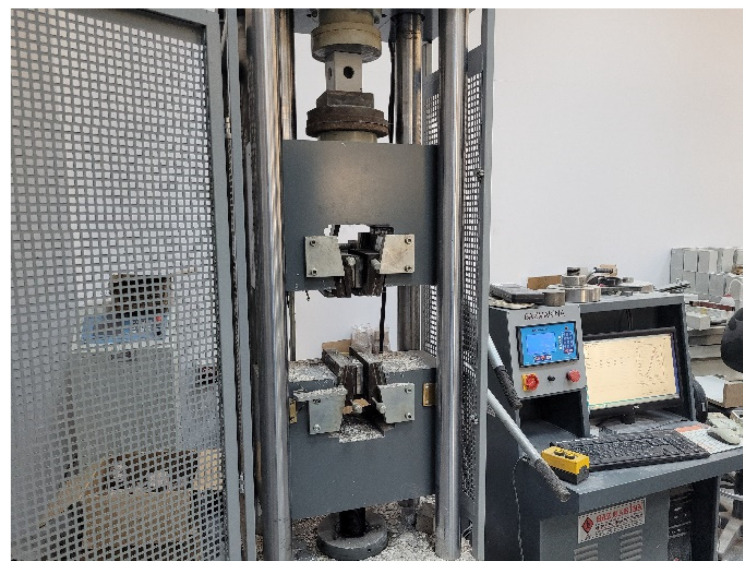
Test setup.

The specimens were tested under compressive loading. The test setup with a capacity of 600 kN, shown in Figure 3, was utilized to perform the tests. The displacements and loads were recorded during the experiments automatically. The specimens were loaded at a speed of 2 kN/s. Three repetitions were performed for each type, and the average of these results was reported.

## 3. Results and Discussion

**Figure 4:**
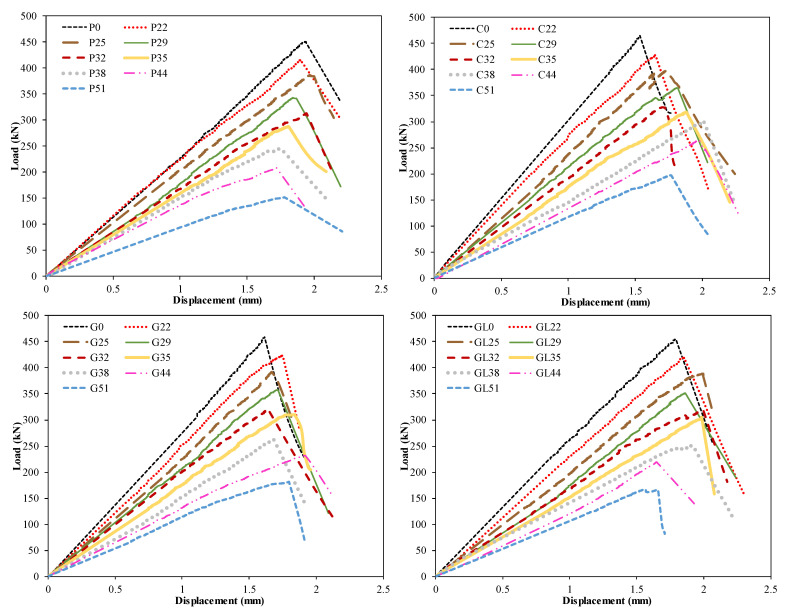
Load–displacement curves of openings with different opening sizes.

The experimental results of 51 specimens with different opening sizes, opening locations, and number of holes are presented. In the results, P represents the pultruded profile without any wrapping, C represents the pultruded profile with CFRP wrapping, G represents the pultruded profile with 1200 gr/m^2^ GFRP, and GL represents the pultruded profile with 300 gr/m^2^ GFRP. The load capacities of these specimens are given in Table 3, while the load–displacement curves are depicted in Figure 4. The load–displacement curves were obtained from the samples, which gave average results among three repetitions.

The failure modes of the pultruded GFRP box sections can be summarized as follows. These observations of the failure modes were closely related to the specimens’ load–displacement responses, which will be discussed in the following section. 

**Figure 5:**
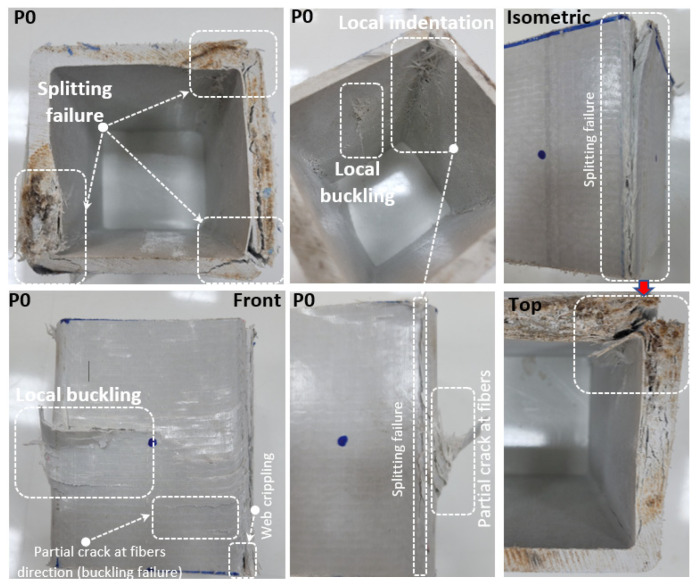
Damage analyses of P0.

First, the pultruded profiles without any strengthening (P) were compared. Here, the hole diameter/width of pultruded profile ratio (D/W) varied between 29% and 68%. When Figure 4 and Table 3 are examined, the decrease in load-carrying capacity of P22, P25, P29, P32, P35, P38, P44, and P51 compared to P0 is 7.9%, 14.22%, 23.90%, 30.67%, 36.32%, 45.56%, 53.88% and 66.44%, respectively. In addition, as the diameter of the holes drilled into the samples increased, the initial stiffness values decreased. This naturally led to a decrease in linear energy consumption capacity. In other words, since no ductile behavior was observed in any of the samples, evaluations could be made based on their linear elastic behavior. Therefore, considering the linear elastic behavior among all samples, the temporarily stored energy consumption was observed at least in the P51 sample. This shows that the elastic energy dissipation capacity of P51 is the least effective under sudden vertical load effects. However, differences in damage observed in samples tested under pressure were also detected. The damages observed in the P22 sample under compression are concentrated especially in the corner areas. The splitting damages observed in the corner areas limited its load-carrying capacity. It is understood that the weakest regions are the corner points. In addition, initial local buckling was observed before splitting damage occurred at the corner points. This situation can be seen more clearly in the damage photographs shown in Figure 5.

**Figure 6:**
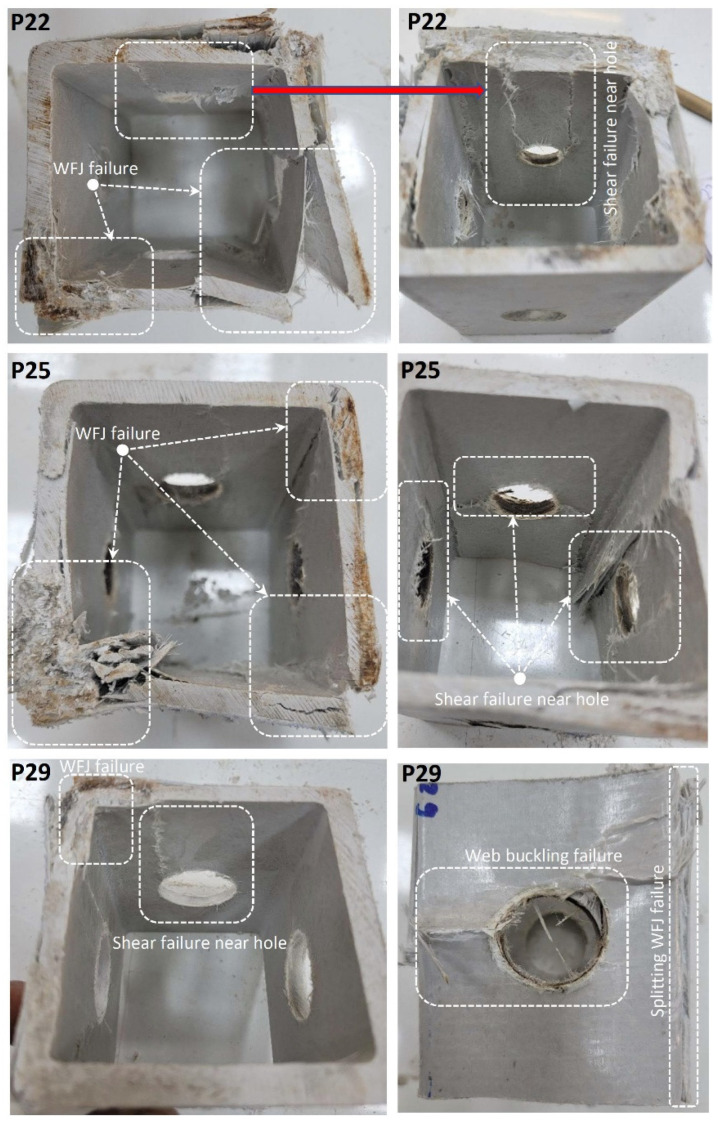
Damage analyses of P22, P25, and P29.

The first samples had holes with 22 mm, 25 mm, 29 mm, and 32 mm diameters opened to the midpoints of the pultruded profile’s four edges and are the P22, P25, P29, and P32 samples. On one hand, the damage behavior of P22, which represents the sample with the smallest hole diameter, and those of P25, P29 and P32 are quite different from P0. The typical failure modes are shown in Figure 6 for box sections of P22, P25, P29, and P32. After the end of the test, splitting occurred at the web–flange junction (WFJ) on the P22 specimen. With the increase in loading, web–flange separation, shear damage around the hole, and the bearing plates cutting into the web were observed. On the other hand, the subsequent failures of P25, P29, and P32 were very similar to that of P22, which was the web–flange separation. In P29, the damage at the web–flange junction was observed in the subsequent loading process of buckling specimens. Finally, the load-carrying capacities of P25, P29, and P32 were 6.84%, 17.3%, and 24.7% less than P22, respectively. However, similar damage behaviors were observed in P22, P25, P29, and P32.

**Figure 7:**
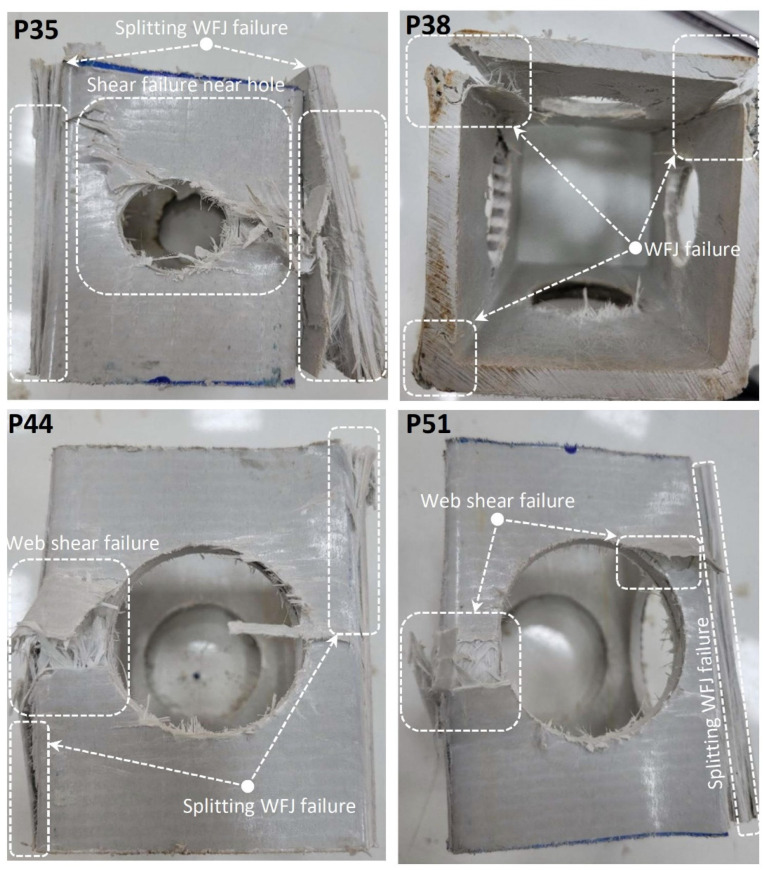
Damage analyses of P35, P38, P44, and P51.

When the P35, P38, P44, and P51 specimens were compared, differences in damage types were observed, coinciding with the increase in hole diameter (Figure 7). While web buckling with cracking at the midpoint was observed in specimens with D/W ratio below 0.46, direct shear damage was observed in samples above this ratio. That is, as the hole diameter increases, the initial failure was 45° cracking at the web–flange junction. This situation was evaluated as the critical threshold for the D/W ratio. An initial cracking at almost 45° at the web–flange junction was experienced on the P35 specimen. While a web–flange junction failure was observed in the corner regions of these four specimens, web shear failure was observed around the hole in the P44 and P51 specimens. It should be noted that, although P35, P38, P44, and P51 showed the same initial web–flange junction failure, the processes of all of these specimens before reaching this initial failure were different because of the D/W ratio.

**Figure 8:**
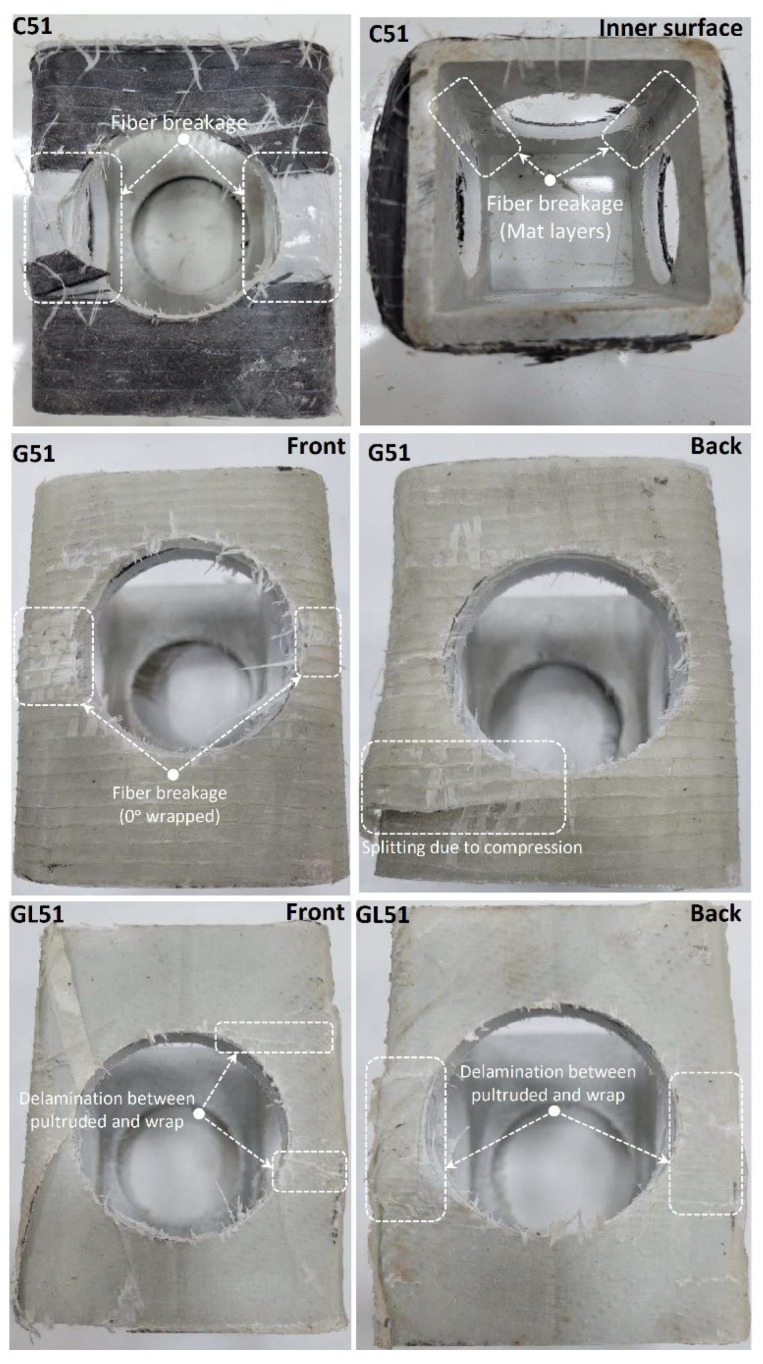
Damage analyses of C51, G51, and GL51.

Following the reference specimens, specimens with each D/W ratio were tested after C-, G-, and GL-type reinforcements. The resulting damages show differences both according to the reinforcement type and according to the reference specimen. WFJ failure, which was first seen in the reference specimen, was prevented in each reinforcement application. This shows that strengthening is effective and that the weaknesses of PGFRP are eliminated. In Figure 8, fiber breakage was observed around the hole in specimens C51 and G51, while additional splitting damage was observed in specimen G51. Delamination damage was observed around the hole in the GL 51 specimen. When these three specimens are compared, the load-carrying capacity of C51 is 8.7% and 17.7% higher than G and GL. It was observed that all three reinforcement treatments prevented WFJ damage, especially in the reference specimen. It was also observed that easier applicability and a better adhesion interface formed between GL and pultruded GFRP.

**Figure 9:**
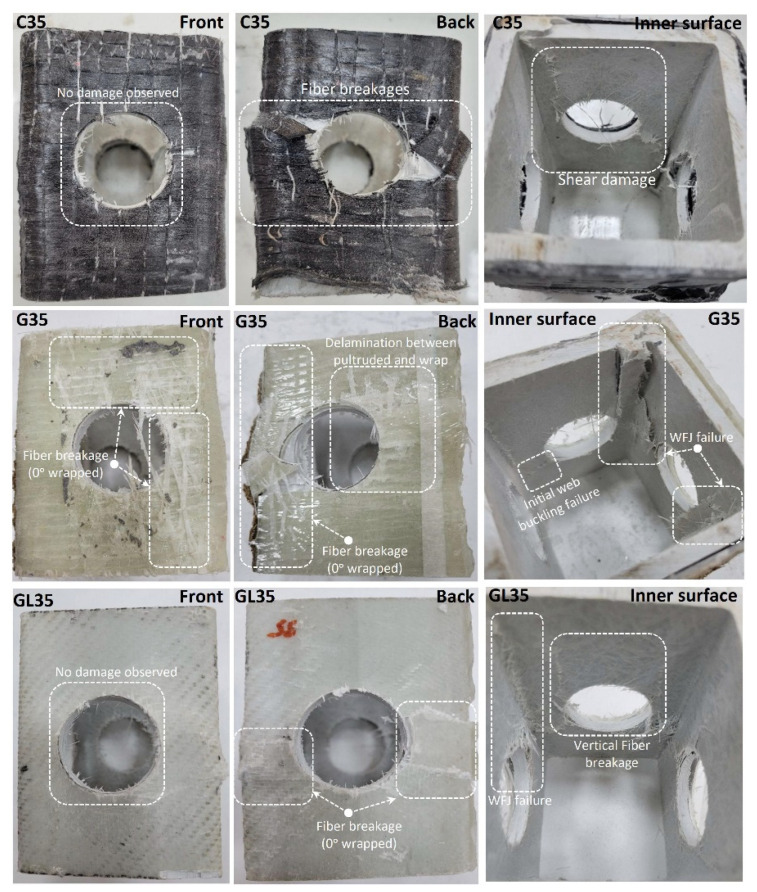
Damage analyses of C35, G35, and GL35.

Damage analysis is not shown, as the damage observed in Figure 8 occurred similarly in C44, G44, and GL44 specimens. Similarly, C44, which had the highest load-carrying capacity, showed increases of 14.7% and 20.9% compared to G44 and GL44. This situation occurred similarly in the C38, G38, and GL38 specimens. Here, the load-carrying capacity of C38 is 14.7% and 19.9% higher than G38 and GL38, respectively. Here, it was determined that the reference specimens with D/W ratios of 0.50 and 0.58 exhibited similar rates of increase after strengthening. In Figure 9, the observed damages in the C35, G35, and GL35 specimens are given. With the strengthening of the specimens with a D/W ratio of 0.46, it was observed that the shear damage around the hole and WFJ observed in the reference specimen was largely prevented. Damages occurring around the hole with the highest stress applied also differed according to the reinforcement type. When the damages on the front and back surfaces of the specimens were examined, it was observed that GL formed a better adhesion interface, resulting in less internal surface damage. Fiber damage observed in C- and G-type reinforcements led to an increase in internal surface damage. However, when the load-carrying capacities are compared, it was determined that C35 increased by 2.4% and 4.1%, respectively, compared to G35 and GL35. Therefore, GL35, which has a relatively lower load-carrying capacity compared to C35, can be said to be effective as a reinforcement alternative since it is easy to apply and has less damage.

**Figure 10:**
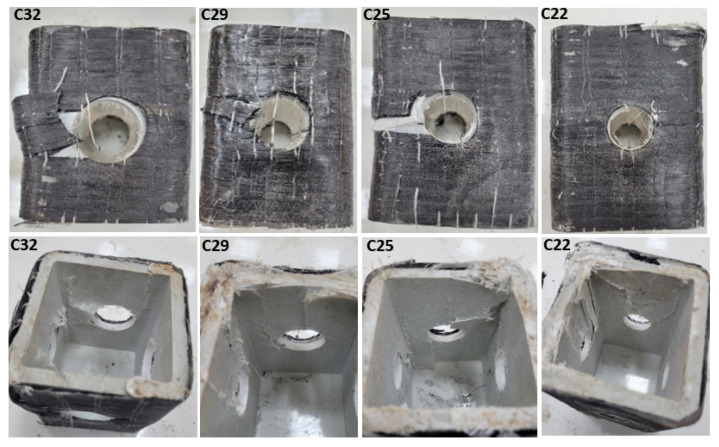
Damage photos of the C32, C29, C25, and C22 specimens.

**Figure 11:**
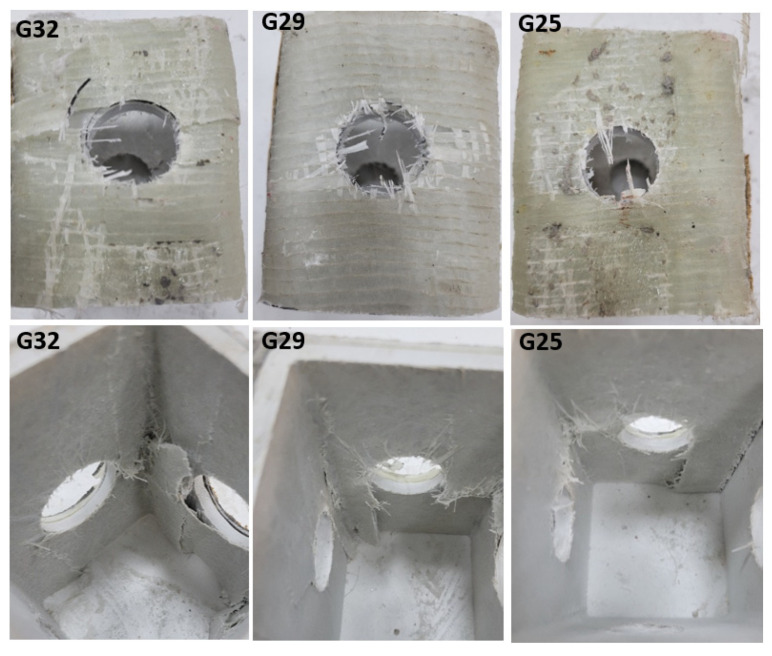
Damage photos of the C32, C29, C25, and C22 specimens.

Similar damages shown in Figure 9 were also observed in specimens with diameters of 32 mm, 29 mm, 25 mm, and 22 mm. For example, the load capacity of the C32 sample increased by 2.1% and 3.1% compared to the G32 and GL32 specimens, respectively. This was observed as similar increases in the 29 mm, 25 mm, and 22 mm specimens. Here, the increase in the load-carrying capacity of the reinforced specimens relative to each other decreases in accordance with the decrease in the hole diameter. In other words, it is thought that a selection should be made by considering the damage types that occur in the selection of the reinforcement alternative of the hole diameter. Therefore, it is understood that the reinforcement alternative that allows less cost, easier applicability, and less damage should be selected. Therefore, it is thought that a GL-type reinforcement may take the first place as the preferred alternative. In Figure 10, images of the damage to the specimens with 32 mm 29 mm, 25 mm, and 22 mm hole diameters reinforced with carbon are given. In Figure 11, images of the end-of-experiment damage of the G32, G29, and G25 specimens are given.

**Figure 12:**
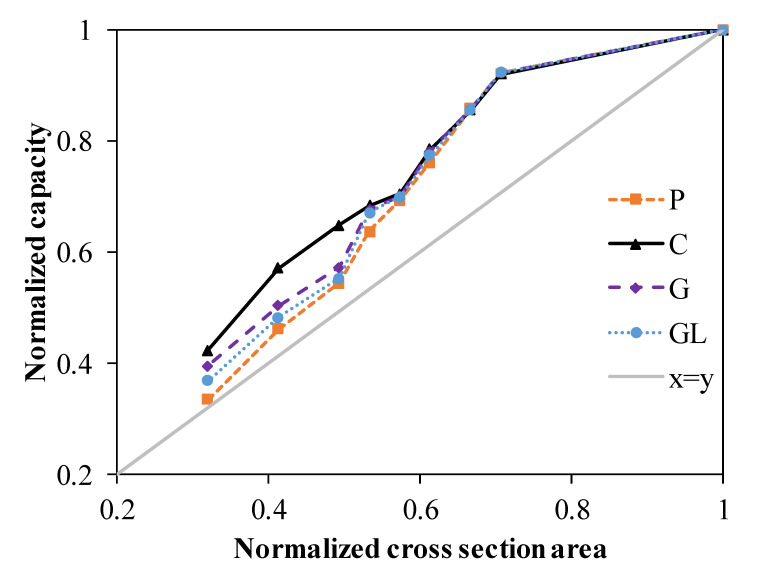
Normalized cross section vs. normalized capacity.

Figure 12 demonstrates the relation between normalized capacity and normalized cross section. The capacity of the specimens with openings was normalized by the capacity of the specimen without an opening, while the cross section with an opening was normalized with the whole cross section. It is seen that pultruded profiles with openings are able to compensate for the reduction due to holes up to a 0.39 reduction in the cross section. As the reduction in the cross-section increases, the capacity significantly drops after a 0.39 reduction in capacity. Moreover, the pultruded profile with CFRP wrapping is more likely to compensate the capacity compared to other wrappings. The observed damage development for each specimen is summarized in Table 4.

## 4. Conclusions

In this study, it has been investigated how the bearing capacities of PGFRP profiles with holes in them change according to various parameters. Web openings of different diameters were opened in the center of the profiles and in different locations of the profile, and these specimens were reinforced by wrapping them with carbon/glass FRP fabrics. The following results were obtained:

- The effect of CFRP and GFRP strengthening for the pultruded box columns, within which the openings were under a compression load, was examined. From the experimental results obtained in the study, it is seen that there is no need to take any precaution for low-strength fibers (300 gr/m^2^ GFRP) used for pultruded columns with openings, provided that the D/W ratios do not exceed 0.68. However, it was observed that the decrease in load-bearing capacity and the decrease in initial stiffness coincided with the increase in the hole diameters. It is observed that the CFRP (C), GFRP (G), and GFRP (GL)-strengthening methods can restore the pultruded column to the reference capacity level, especially for the columns where the D/W ratio is between 0.29 and 0.68. It can be concluded that all strengthening applications will be effective up to a certain D/W ratio. After the experiment, the damage modes occurring in CFRP (C), GFRP (G), and GFRP (GL) applications were detected in the micro damage analysis and ranked according to the order of damage development. According to this analysis, the damages that occurred were fiber bundles debonding, delamination between fiber and pultruded column in the direction of the fiber, splitting, web–flange junction failure, and fiber breakages.
- The pultruded profiles with openings can compensate the reduction in load-bearing capacity with holes up to a 0.39 D/W ratio. The capacity significantly drops after a D/W ratio of 0.39. Moreover, the pultruded profile with CFRP wrapping is more likely to compensate the capacity compared to other wrappings.

## Figures and Tables

**Table 1:** Mechanical properties of pultruded GFRP.

Property	Mean Value (MPa)
Longitudinal tensile modulus of elasticity	23,000
Transverse tensile modulus of elasticity	7000
Longitudinal tensile strength	240
Transverse tensile strength	50
Longitudinal compressive strength	150
Transverse compressive strength	70
Shear strength	25

**Table 2:** Material properties of FRP fabrics.

CFRP Strip Properties (800 gr/m^2^)	Values
Thickness (mm)	0.85
Tensile strength (GPa)	4.4
Modulus of elasticity (GPa)	235
Rupture strain (%)	1.87
**GFRP strip properties (1200 gr/m^2^)**	**Values**
Thickness (mm)	1.2
Tensile strength (GPa)	3.5
Modulus of elasticity (GPa)	80
Rupture strain (%)	4.37
**GFRP strip properties (300 gr/m^2^)**	**Values**
Thickness (mm)	0.3
Tensile strength (GPa)	0.586
Modulus of elasticity (GPa)	31.2
Rupture strain (%)	1.91
**Epoxy + Hardener (F-1564 + F-3486)**	**Values**
Tensile strength (GPa)	0.055
Modulus of elasticity (GPa)	2.090
Rupture strain (%)	4.06 ± 1.27

**Table 3:** Results of the specimens with different hole sizes.

Hole Size	D/W	P (kN)	Reduct. %	C (kN)	Reduct. %	G (kN)	Reduct. %	GL (kN)	Reduct. %
0	0	450.6	1	464.9	1	458.6	1	454.5	1
22	0.29	414.9	7.9	427.5	8.04	422.8	7.8	418.9	7.8
25	0.32	386.5	14.22	397.1	14.58	392.5	14.41	389.3	14.34
29	0.39	342.9	23.90	364.9	21.51	357.6	22.02	351.1	22.75
32	0.42	312.4	30.67	327.6	29.53	320.8	30.04	317.6	30.12
35	0.46	286.9	36.32	317.8	31.64	310.1	32.38	305.1	32.87
38	0.50	245.3	45.56	301.1	35.23	262.3	42.80	251.1	44.75
44	0.58	207.8	53.88	265.1	42.97	231	49.63	219.2	51.77
51	0.68	151.2	66.44	196.9	57.64	181.1	60.51	167.3	63.19

D: Hole diameter; W: Pultruded box width.

**Table 4:** Summary of the damages observed in the experiments.

Test Specimens	Damage Modes	Explanation
P0	Splitting failure Local buckling Local indentation Web Crippling	Splitting failure was observed on web–flange junction. In addition, local buckling occurred in the middle of the profile height, with the effect of compression. Local indentation was observed in the upper region where the load was applied, and web crippling (crushing behavior) was observed in the lower region.
P22-P25-P29-P32	WFJ failure Shear failure near hole Web buckling failure	Shear damage was observed starting around the hole and progressing to the profile height. In addition, splitting damage occurred at the profile corner points. Web buckling failure with shear damage around the hole was observed in P29.
P35-P38-P44-P51	WFJ failure Web shear failure Shear failure near hole	While web buckling with cracking at the mid was observed in specimens with a D/W ratio below 0.46 (P35 specimen), direct shear damage was observed in specimens above this ratio.
C51-G51-GL51	Fiber breakage Splitting due to compression Delamination between pultruded and wrap	WFJ failure prevented in each strengthening application. Fiber breakage was observed around the hole in specimens C51 and G51, while additional splitting damage was observed in specimen G51. Moreover, delamination was observed around the hole in the GL51.
C35-G35-GL35	Fiber breakage Shear damage near hole Delamination between pultruded and wrap	GL formed a better adhesion interface, resulting in less internal surface damage. Fiber damage observed in C- and G-type reinforcements led to an increase in internal surface damage.

## Data Availability

Not applicable.
